# Vibro-Tactile Enhancement of Speech Intelligibility in Multi-talker Noise for Simulated Cochlear Implant Listening

**DOI:** 10.1177/2331216518797838

**Published:** 2018-09-16

**Authors:** Mark D. Fletcher, Sean R. Mills, Tobias Goehring

**Affiliations:** 1 Institute of Sound and Vibration Research, Faculty of Engineering and Physical Science, University of Southampton, UK; 2 MRC Cognition and Brain Sciences Unit, University of Cambridge, UK

**Keywords:** multisensory, touch perception, speech perception

## Abstract

Many cochlear implant (CI) users achieve excellent speech understanding in acoustically quiet conditions but most perform poorly in the presence of background noise. An important contributor to this poor speech-in-noise performance is the limited transmission of low-frequency sound information through CIs. Recent work has suggested that tactile presentation of this low-frequency sound information could be used to improve speech-in-noise performance for CI users. Building on this work, we investigated whether vibro-tactile stimulation can improve speech intelligibility in multi-talker noise. The signal used for tactile stimulation was derived from the speech-in-noise using a computationally inexpensive algorithm. Eight normal-hearing participants listened to CI simulated speech-in-noise both with and without concurrent tactile stimulation of their fingertip. Participants' speech recognition performance was assessed before and after a training regime, which took place over 3 consecutive days and totaled around 30 min of exposure to CI-simulated speech-in-noise with concurrent tactile stimulation. Tactile stimulation was found to improve the intelligibility of speech in multi-talker noise, and this improvement was found to increase in size after training. Presentation of such tactile stimulation could be achieved by a compact, portable device and offer an inexpensive and noninvasive means for improving speech-in-noise performance in CI users.

## Introduction

Many cochlear implant (CI) users achieve excellent speech understanding in acoustically quiet conditions (Fetterman & Domico, 2002; Zeng, Rebscher, Harrison, Sun, & Feng, 2008), but most, even with state-of-the-art implants, perform poorly in the presence of background noise (Spriet et al., 2007; Wouters & Van den Berghe, 2001). An important contributing factor to this poor speech-in-noise performance is the limited transmission of low-frequency sound information through CIs. This has been demonstrated by studies in normal-hearing subjects listening to CI simulations (NHCIs), which have shown that the addition of unprocessed low-frequency sound improves speech-in-noise performance (Chang, Bai, & Zeng, 2006; Qin & Oxenham, 2006). Studies have also shown improved speech-in-noise performance, as well as other benefits such as improved sound localization and music perception, in CI users who retain residual low-frequency acoustic hearing (O'Connell, Dedmon, & Haynes, 2017). Unfortunately, few patients referred for CI fitting have usable residual hearing (Verschuur, Hellier, & Teo, 2016).

The low-frequency sound that has been found to improve speech-in-noise performance in some CI users is within a frequency range of around 20 Hz to 500 Hz (Verschuur et al., 2016). This matches the frequency range in which the tactile system is most sensitive (Verrillo, 1963). Traditionally, researchers have used tactile aids to support speech perception in people with severe hearing impairment as an alternative to CIs but with limited success (e.g., Hnath-Chisolm & Kishon-Rabin, 1988; Sherrick, 1984; Weisenberger, 1989). More recently, Huang, Sheffield, Lin, and Zeng (2017) showed that speech-in-noise performance in CI users can be improved by presenting the fundamental frequency (F0) of the speech signal via vibro-tactile stimulation. However, some aspects of Huang et al.'s approach limit its real-world applicability, namely: (a) that the tactile signal was extracted from the clean speech rather than from the speech-in-noise signal, as would be required in a real-world application, and (b) that stationary background noise was used to assess speech-in-noise performance rather than multi-talker babble noise, in which CI users struggle most (Oxenham & Kreft, 2014; Zeng et al., 2008).

The primary aim of this study was to determine whether tactile stimulation can improve speech intelligibility in multi-talker noise for NHCIs, when the tactile signal is derived from the speech-in-noise signal. The signal processing approach used in this study extracted the temporal envelope and voicing information, which have been shown to provide similar benefit to F0 in acoustic presentation for NHCIs (Brown & Bacon, 2009; Kong & Carlyon, 2007). These were then used to modulate seven low-frequency carrier tones which were at frequencies where touch perception is most sensitive. The envelope modulations were amplified using an expander function, which was intended to increase the saliency of the speech envelope and reduce the contribution from background noise. The approach used in this study is less computationally intensive than F0 extraction and may be more appropriate for real-time application. Furthermore, as discussed by Carroll, Tiaden, and Zeng (2011), accurate real-time F0 extraction may not be feasible in real-world situations with multi-talker noise, and recent work has shown that F0 extraction errors increase rapidly at signal-to-noise ratios (SNRs) below 10 dB (Jouvet & Laprie, 2017).

The secondary aim of this study was to establish whether any tactile enhancement of speech-in-noise performance becomes larger after training. To establish this, speech-in-noise performance for NHCIs was measured with and without tactile stimulation both before and after a 3-day training regime in which participants were exposed to concurrent speech-in-noise and tactile stimulation. An increase in tactile enhancement after training was anticipated, as previous studies using tactile aids to improve speech intelligibility in deaf and hearing-impaired individuals without a CI have found large increases in performance with training (Brooks, Frost, Mason, & Gibson, 1986a, 1986b; Sherrick, 1984; Weisenberger, Heidbreder, & Miller, 1987).

## Methods

### Participants

Eight participants (five men and three women, aged between 22 and 29 years old) were recruited from the staff and students of the University of Southampton, and from acquaintances of the researchers. Participants were not paid for their participation. All participants reported no hearing or touch issues on a screening questionnaire (see Appendix). They were also assessed by otoscopy and pure-tone audiometry. Participants had hearing thresholds not exceeding 20 dB hearing level (HL) at any of the standard audiometric frequencies between 0.25 and 8 kHz in either ear. Participants also had their vibro-tactile thresholds measured (see Procedure section). All participants had thresholds below 0.3 ms^−2^ root-mean-square (RMS) at 31.5 Hz and 0.7 ms^−2^ RMS at 125 Hz, indicating normal touch perception (ISO 13091-2:2003, 2003). Participant characteristics are shown in Table 1.

**Table 1.** Summary of Participant Characteristics. Individual Data as Well as the Mean and Standard Error Across Participants are Reported.

Participant	Gender	Age	Dominant hand	Vibro-tactile threshold at 31.5 Hz (ms^−2^ RMS)	Vibro-tactile threshold at 125 Hz (ms^−2^ RMS)
1	M	28	R	0.11	0.33
2	F	29	R	0.06	0.14
3	M	26	R	0.07	0.08
4	M	25	R	0.12	0.30
5	F	23	R	0.08	0.14
6	M	23	R	0.06	0.18
7	M	22	R	0.17	0.11
8	F	28	R	0.16	0.07
Mean		25.5		0.10	0.17
SE		0.95		0.02	0.03

*Note*. M = male; F = female; RMS = root-mean-square; R = right.

### Cochlear Implant Simulation and Tactile Signal Generation

Acoustic signals processed with noise or tone vocoders have been used to simulate speech perception with CIs in several studies (Dorman, Loizou, Fitzke, & Tu, 1998; Shannon, Zeng, Kamath, Wygonski, & Ekelid, 1995; Qin & Oxenham, 2003). In this study, we used the SPIRAL vocoder for CI simulation, which has recently been developed to achieve a more accurate simulation of the effects of current spread in the cochlea (Grange, Culling, Harris, & Bergfeld, 2017). The speech reception scores for normal-hearing participants better match those of CI users when the SPIRAL vocoder is used than when a traditional noise-band vocoder is used (Grange et al., 2017).

**Figure 1.**
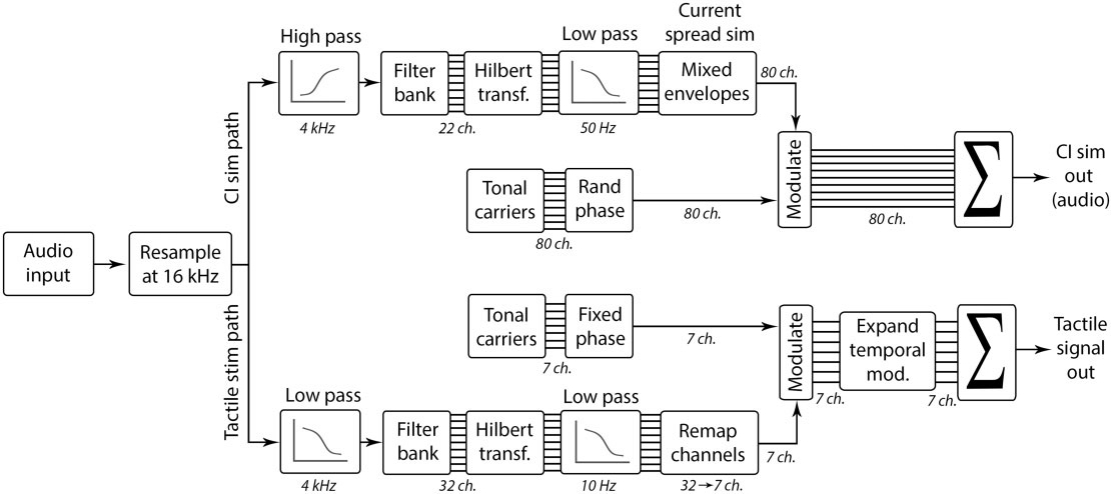
Schematic representation of the signal processing chain for the cochlear implant simulation (upper signal processing path) and tactile signal generation (lower signal processing path). CI = cochlear implant.

Figure 1 illustrates the signal processing chain. To generate the CI simulations, the audio signal was resampled with a sampling frequency of 16 kHz and then passed through a first-order high-pass filter with a cutoff frequency of 4 kHz, similar to the input filter characteristics applied in CI speech processors (Chung & McKibben, 2011). The signal was then passed through an FIR filter bank with 22 center frequencies ranging from 250 to 8000 Hz, equally spaced on the equivalent rectangular bandwidth (ERB) scale (Glasberg & Moore, 1990). These 22 filter channels represent the 22 electrodes on an implanted electrode array in the inner ear of a CI user, with the number of simulated electrodes chosen to be the same as with implants produced by the manufacturer Cochlear Ltd. (Sydney, Australia). Following Grange et al. (2017), the envelopes of each channel of the filter bank were computed by calculating the Hilbert transform and applying a first-order low-pass filter with a cutoff frequency of 50 Hz. An envelope mixing function was then used to obtain a sum of weighted contributions from each simulated electrode channel to simulate the spread of excitation in the cochlea. Eighty tonal random-phase carriers were generated in the frequency range from 300 to 8000 Hz (with ERB spacing) and were modulated by the mixed envelopes. The envelope information was applied to the tonal carriers as a representation of the neural excitation patterns of electrically stimulated spiral ganglion cells. The default value of 8 dB per octave for the current decay slope was used, in line with tuning curve slopes measured using monopolar stimulation in CI users (Nelson, Kreft, Anderson, & Donaldson, 2011). The tonal carriers were then summed up to form the CI simulation output signal for acoustic presentation to the participant.

**Figure 2.**
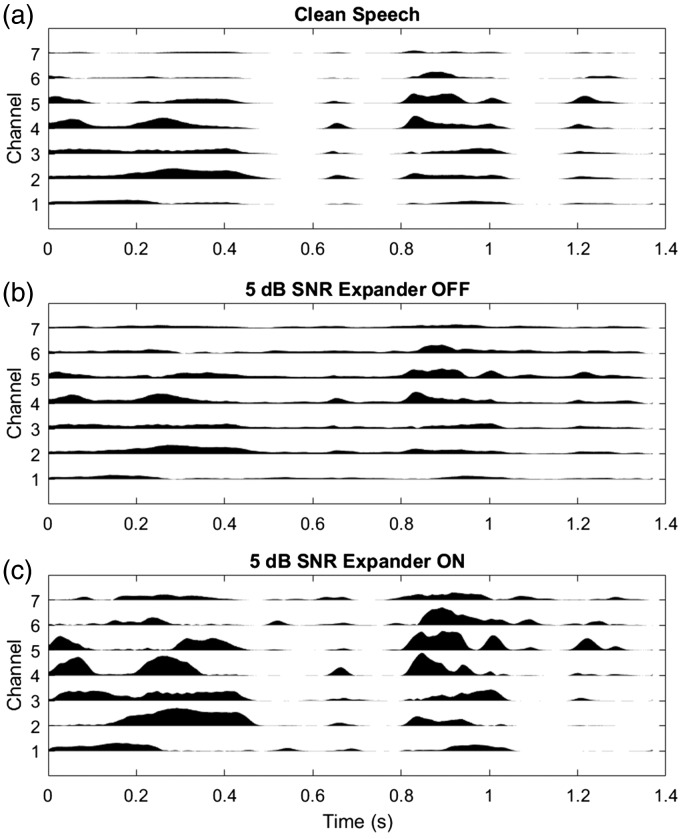
Illustration of the effect of the expander on the tactile signal. Panel A shows the tactile signal for clean speech (with the expander turned off), Panel B shows the tactile signal for speech mixed with multi-talker noise at an SNR of 5 dB (the lowest SNR used in this study was 5.8 dB) with the expander turned off, and Panel C shows the same signal as Panel B, but with the expander turned on. The amplitude envelopes for each of the seven frequency channels of the tactile signal for the sentence “They moved the furniture” spoken by a male speaker (BKB sentence corpus) are shown in each panel. The height of each channel waveform corresponds to the amplitude of the signal. SNR = signal-to-noise ratio.

To generate the tactile signal, the audio input signal was resampled with a sampling frequency of 16 kHz, and a first-order low-pass filter with a cutoff frequency of 4 kHz was applied. The low-pass filter was applied, first, to attenuate high frequency information that is efficiently transmitted by a CI and, second, to keep the signal in sync with the acoustic path by imposing the same processing delay. The signal was then passed through an FIR filter bank with 32 channels with center frequencies ranging from 100 to 1000 Hz, equally spaced on the ERB scale, which yields a higher concentration of channels at lower frequencies. This frequency range was selected to include the frequencies most dominant in speech (Byrne et al., 1994). For each channel of the filter bank, the Hilbert envelope was computed, and a first-order low-pass filter was applied with a cutoff frequency of 10 Hz. This low-pass filter limited the modulation frequency range to between about 1 and 30 Hz, which is the range most important for speech intelligibility (Drullman, Festen, & Plomp, 1994). The 32 channels were linearly remapped to seven channels (by resampling in the frequency domain) and used to modulate the amplitude envelopes of seven tonal carriers with center frequencies ranging from 30 to 300 Hz (a frequency range in which the tactile system is highly sensitive; Verrillo, 1963). The carriers had a 45-Hz frequency spacing and fixed phases. These carriers were chosen because they would be expected to be individually discriminable based on estimates of vibro-tactile frequency difference limens (Rothenberg, Verrillo, Zahorian, Brachman, & Bolanowski, 1977), although the results of some studies have suggested that information transfer for complex signals is more limited when these signals are summed and presented to a single site (Israr, Tan, & Reed, 2006; Rabinowitz, Houtsma, Durlach, & Delhorne, 1987; Summers et al., 1997). Each of the seven modulated carrier signals was individually passed through an expander function (which was based on Zölzer, 2011) to amplify temporal modulations, and thereby increase the saliency of speech envelope information, and to reduce the contribution from the multi-talker background noise. Figure 2 illustrates the effect of the expander, with Panel A showing the processed clean speech (without the expander) and Panels B and C showing the processed speech in multi-talker noise at 5 dB SNR with and without the expander. The expander function applied additional gain to enhance fluctuations in the amplitude of each channel with a maximum amplification of 6 dB, attack and release times of 10 and 100 ms, a slope of 6 dB per octave, and a threshold set to the RMS level of the signal. The enhanced tonal carriers were then summed up to form the input signal for tactile presentation to the participant. The tactile signal was presented through a *HVLab* tactile vibrometer. The mean amplitude for a single sentence was 1.96 ms^−2^ RMS.

### Speech and Noise Stimuli

Two different speech corpora were used in this study. The Bamford-Kowal-Bench (BKB) Institute of Hearing Research male sentence corpus was used for speech testing. Training and familiarization were conducted using speech material from the RealSpeech™ (United Kingdom) content library (used with permission of Dr. Ian Wiggins and Dr. Mark Fletcher), which used different talkers than the BKB sentence corpus. RealSpeech material was recorded under near-anechoic conditions and comprises a set of narratives that cover a variety of general-interest topics. For both training and speech testing, a nonstationary multi-talker noise recorded by the National Acoustic Laboratories (NAL; Keidser et al., 2002) was used. The noise was a real-world recording made at a party, with a spectrum that matched the international long-term average speech spectrum (Byrne et al., 1994). All speech-in-noise material was processed for audio presentation using a CI simulation based on vocoder processing and was also processed separately for tactile presentation (see earlier section).

### Equipment

All stimuli were generated and controlled using custom MATLAB scripts (version R2016a, The MathWorks Inc., Natick, MA, USA). During pure-tone audiometry, participants were seated in a sound-attenuated booth with a background noise level conforming to British Society of Audiology (2017) recommendations. Acoustic stimuli were generated by a laptop located in a separate observation room and played out via an RME Babyface Pro soundcard (sample rate of 96 kHz and bit depth of 24 bits) and Sennheiser HDA 200 circumaural, closed-back headphones. The stimuli were calibrated using a Bruel and Kjaer (B&K) artificial ear (Type 4152) with a flat-plate adaptor (DB0843). For calibration, the two earphones were separated by approximately 145 mm, as specified in ISO 389-5:2006 (2006) and the headband tension complied with the requirement of ISO 389-5:2006. Vibro-tactile threshold measurements were made using a *HVLab* Vibro-tactile Perception Meter with a 6-mm contactor with a rigid surround and a constant upward force of 2 N, following the specifications of ISO 13091-1:2001 (2001). The tactile system for the testing and training sessions and for vibro-tactile threshold measurements was calibrated using a B&K calibration exciter (Type 4294).

In testing and training sessions, stimuli were played out via an RME Fireface UC soundcard (Haimhausen, Germany) and ER-2 insert earphones (Etymotic, IL, USA). Stimuli were calibrated using a B&K 2260 Investigator and 4157 occluded ear coupler (Royston, Hertfordshire, UK). The experiment took place in a quiet room. The experimenter sat behind a screen with no line of site to the participant and listened to the signal that was delivered to the participant using Sennheiser HD 380 Pro circumaural, closed-back headphones in order to mask any auditory cues that might unblind the experimenter to the experimental condition. The vibration signal was delivered to the participant via a *HVLab* Tactile Vibrometer with a 10-mm contacting probe to the distal phalanx of the index finger of the participant's right hand (which in all cases was their dominant hand) with an upward force of 2 N.

### Procedure

**Figure 3.**
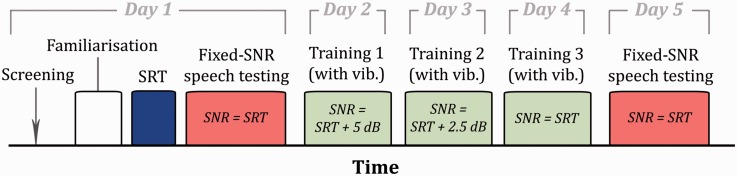
Schematic (not to scale) showing the timeline of the experiment. SNR = signal-to-noise ratio; SRT = speech reception threshold.

Figure 3 shows a schematic illustrating the experimental procedure. On the first of 5 consecutive days, participants were screened (see Participants section) and were then familiarized with speech in quiet processed using the CI simulator without concurrent tactile stimulation. Each participant's speech reception threshold (SRT; the SNR at which 50% performance is obtained) was then measured without tactile stimulation. This SRT was then used as the SNR for speech-in-noise testing in conditions with and without tactile stimulation. On each of the following 3 days, participants were trained with concurrent tactile stimulation, at SNRs that decreased each day. On the fifth day, the speech-in-noise testing was again conducted with and without tactile stimulation, with the SNR fixed to the SRT measured on day 1. Two different speech corpora were used, one for the familiarization and training phases, and one for the SRT and speech testing.

In the screening phase, pure-tone audiometry was conducted following the recommended procedure of the British Society of Audiology (2017). Vibro-tactile detection thresholds were measured using conditions and criteria specified in ISO 13091-1:2001 and ISO 13091-2:2003. These thresholds were estimated for sinusoidal vibrations of 31.5 and 125 Hz using the von Bekésy method of limits. In this procedure, the amplitude of the stimulus increased until the participant pressed a button to indicate they could feel the vibration, at which point the amplitude decreased until the participant could no longer feel the vibration. The amplitude changed by 5 dB/s for the first two reversals, and then by 3 dB/s for the remaining eight reversals that made up the threshold track. The threshold was taken as the average of the last six reversals. For each frequency, the procedure was conducted twice, and the mean taken as the threshold.

Following the screening phase, participants were familiarized with CI simulated speech (in quiet and with no tactile stimulation) using a 5-min speech segment from a male talker from the RealSpeech content library (see Speech and Noise Stimuli section). Participants were given a transcript of the speech with some sections of the text blacked out and were asked to report to the experimenter what was said in the missing sections. This phase allowed participants to become comfortable with the unusual sound of the CI simulated speech.

After the familiarization phase, each participant's SRT was measured using a single BKB sentence list (containing 15 sentences) mixed with multi-talker noise. The SNR of the first trial was 5 dB. The sentence used in the first trial was repeated, with the SNR increased by 2 dB after each repeat, until the participant got at least two out of three keywords correct. A one-up one-down adaptive tracking procedure (Levitt, 1971) with a step size of 2 dB was then followed for the remaining 14 sentences (tracking 50% correct performance). The speech signal was always presented at a level of 65 dB SPL LAeq. The SRT was calculated as the mean of the last six reversals. Two SRT estimates were made for each participant. The average SRT across participants was 7.9 dB (ranging from 5.8 to 14 dB), which is similar to the mean and range typically seen in CI users (e.g., Goehring et al., 2017).

In the speech testing phases before and after the training, the percentage of keywords correctly reported was measured. Two sets of eight BKB sentence lists were used. Which of the sets was used for pre-training and which for post-training was counterbalanced across participants. In each speech testing phase, four of the sentence lists were used to measure performance in the condition with tactile stimulation, and four in the condition without tactile stimulation. The two conditions were alternated in an A-B-A-B pattern across the lists. Whether tactile stimulation was applied in Condition A or B was counterbalanced across participants, such that half of the participants had tactile stimulation in Condition A and half in Condition B for all testing sessions. The experimenter was blinded to whether the participant was receiving tactile stimulation to avoid experimenter bias (see Equipment section). The participant was either instructed via a text display to place their finger on a shaker contact, with the message “Vibration enhancement ON. Audio enhancement OFF.” displayed, or was instructed to put both hands on their lap, with the message “Vibration enhancement OFF. Audio enhancement ON.” displayed. This latter message falsely stated that the audio signal had been enhanced in the condition without tactile stimulation. This false cue was included to control for effects of participant expectation that tactile stimulation was intended to improve performance. Performance was scored as the percentage of correctly reported keywords.

In the training sessions, the target speech consisted of six speech segments from the RealSpeech content library each lasting around 5 min, which were passed through the CI simulation. Half of the segments were read by female talkers and half by male talkers. The segments were split into single sentences and mixed with the NAL multi-talker noise. Participants were asked to repeat each sentence to the experimenter, after which the sentence text was displayed to the participant. In each session, two segments (totaling around 10 mins) were presented. The order in which the speech segments were presented was randomized across participants. The task was made more difficult in each successive training session. In the first training session, the SNR was set at 5 dB above the participant's SRT, in the second at 2.5 dB above, and in the final session at the participant's SRT. For all training material, concurrent tactile stimulation was provided.

The experimental protocol was approved by the University of Southampton Ethics Committee (ID: 30753).

## Results

**Figure 4.**
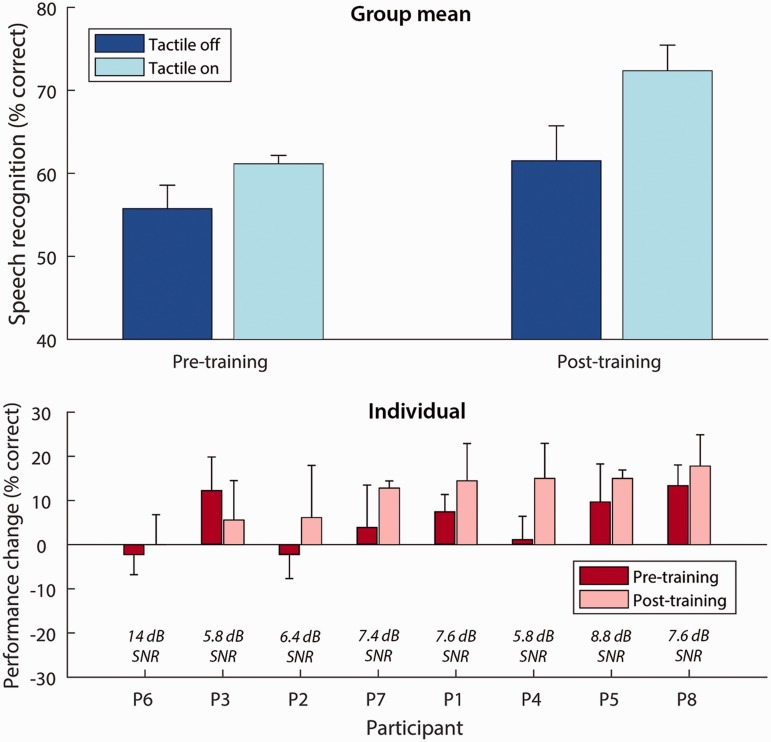
Mean speech-in-noise performance across all participants with and without tactile stimulation before and after training (top panel) and for each individual ordered by the size of their post-training performance change (bottom panel). The SNR at which speech-in-noise performance was measured is shown on the bottom panel for each individual. Error bars show the standard error of the mean. SNR = signal-to-noise ratio.

Figure 4 shows the effect of tactile stimulation on speech-in-noise performance (the percentage of keywords correctly identified) before and after training. The results were analyzed using a repeated-measures analysis of variance, with factors “Session” (before or after training) and “Condition” (with or without tactile stimulation). A significant main effect of condition was measured, *F*(1, 7) = 18.0, *p* = .004, $\eta_{\text{p}}^{2}$ = .72, such that a greater percentage of keywords were correctly identified in the condition with tactile stimulation than without. A significant interaction between session and condition was found, indicating that the effect of tactile stimulation in the post-training session was significantly larger than in the pre-training session, *F*(1, 7) = 6.6, *p* = .037, $\eta_{\text{p}}^{2}$ = .48. Paired *t*-tests (with a Bonferroni corrected alpha of .0125) revealed a significant effect of condition in the post-training session, *t*(7) = 5.0, *p* = .002, but not in the pre-training session, *t*(7) = 2.5, *p* = .043. The mean effect of tactile stimulation before training was 5.4% (improving from 55.7% without tactile stimulation to 61.1% with tactile stimulation; standard error of the mean: ± 2.2%) and the mean effect of tactile stimulation after training was 10.8% (improving from 61.5% to 72.3%; ± 2.2%). The largest individual effect of tactile stimulation on performance was 17.8% (P8, post-training), and the largest reduction in performance was 2.2% (P2 and P6, pre-training). Evidence of an effect of session was seen in the condition with tactile stimulation, *t*(7) = 4.3, *p* = .004, but not in the condition without tactile stimulation, *t*(7) = 2.0, *p* = .082. An overall effect of session was also observed, *F*(1, 7) = 11.4, *p* = .012, $\eta_{\text{p}}^{2}$ = .62.

## Discussion

In this study, tactile presentation of envelope and voicing information was found to significantly improve the intelligibility of speech in multi-talker noise for NHCIs. After training, tactile stimulation improved the percentage of keywords correctly reported for sentences in noise by 10.8% on average. This is similar to the speech-in-noise performance benefit provided by residual low-frequency acoustic hearing in CI users (Gifford et al., 2013, 2017). Our results build on the work of Huang et al. (2017), who found evidence that tactile stimulation could improve speech-in-noise performance for CI users. Like in this study, Huang et al. found robust effects, though the size of the benefit is difficult to compare directly because of the different outcome measures and speech corpora used. Huang et al. presented tactile signals derived from clean speech, whereas in this study, the tactile signal was derived from speech-in-noise, as would be required in a real-world application. This study also adds to the work of Huang et al., who showed tactile benefit in stationary noise, by showing benefit in multi-talker noise, in which CI users struggle most (Oxenham & Kreft, 2014; Zeng et al., 2008). Taken together, these findings indicate that tactile stimulation has strong potential as a means of improving speech-in-noise performance for CI listeners. It could offer a viable alternative for the majority of CI users who do not benefit from residual low-frequency hearing.

In this study, tactile enhancement of speech-in-noise performance increased in size after just 30 min of exposure to speech-in-noise and tactile stimulation over 3 days. Over this short period, participants were trained by performing a speech-in-noise task while receiving additional speech information through vibration on the fingertip. Participants were trained in this condition only, which could have created a bias towards the condition with tactile stimulation. Further work is needed to establish the most effective training method and how much training is required for maximum performance to be achieved. Previous studies using tactile aids (with no accompanying CI signal) suggest a training period of several months or even years is required to achieve maximum benefit (e.g., Brooks et al., 1986a, 1986b; Sherrick, 1984; Weisenberger et al., 1987). This raises the intriguing possibility that prolonged training could lead to even greater performance enhancements than were observed in this study.

The robust improvement in speech intelligibility by tactile stimulation was achieved for speech in multi-talker noise, and with computationally nonintensive processing that could be applied in real time. Noise-reduction algorithms for CIs have facilitated substantial improvements in speech intelligibility in stationary noise. However, they have struggled to produce similar improvements for multi-talker background noise when no *a priori* information about the target speaker is available (Dawson, Mauger, & Hersbach, 2011; Goehring et al., 2017). These algorithms are typically computationally more intensive than the one proposed in this study and may require an increase in computational resources for integration into CI speech processors.

The effect of tactile stimulation on speech-in-noise performance was assessed at SNRs corresponding to typical SRTs for CI users, which are higher than those for hearing-aid users or normal-hearing listeners. Drullman and Bronkhorst (2004) have shown that speech-in-noise performance for normal-hearing listeners can also be improved by tactile stimulation. They found benefits of tactile stimulation for speech with one or two interfering talkers but not for speech with several interfering talkers. However, as in Huang et al. (2017), Drullman and Bronkhorst presented tactile signals derived from clean speech rather than from the speech-in-noise signal. Further work is required to establish whether the approach used in this study is effective at lower SNRs.

An important limitation of this study is that vibro-tactile stimulation was delivered to the fingertip, which may not be a suitable site for real-world application. Previously, researchers using tactile aids (with no accompanying CI signal) have successfully transferred complex auditory information at the wrist (Weisenberger, 1989), forearm (Hnath-Chisolm & Kishon-Rabin, 1988), and abdomen (Weisenberger & Broadstone, 1989). It is therefore considered likely that tactile enhancement of speech-in-noise performance for CI users can be achieved at sites other than the fingertip. The wrist is a particularly promising candidate for future research as, although it has higher vibro-tactile detection thresholds than the fingertip, researchers have shown that it has similar sensitivity to frequency and amplitude differences (Summers & Whybrow, 2005). Tactile stimulation could be delivered via multiple contacts to maximize information transfer capacity, as has been done previously with tactile aids to transfer more spectral information and even to transfer spatial hearing cues (Richardson & Frost, 1979).

A second limitation was the use of NHCIs rather than actual CI users. CI simulations are an established way of presenting signals with a similar amount of usable information as is obtained by CI users. In this study, the measured SRTs for NHCIs were well matched to those measured in real CI users (e.g., Goehring et al., 2017). The CI simulation used here models channel interactions and current spread present in real CIs, making it more realistic than simple vocoder simulations (Grange et al., 2017). This simulation reproduces the signal received by a CI user with an ideally fitted implant, for which all electrodes are functioning optimally, which is not always achieved in practice. It is possible that real CI users, who may receive more limited auditory information through their CI, will benefit more from the tactile stimulation used in this study.

There are a number of potential benefits of tactile stimulation to CI listening beyond improvements in speech-in-noise performance that should be explored in future work. These include the additional benefits that are provided by residual low-frequency acoustic hearing to CI users, such as enhanced music perception and spatial hearing (O'Connell et al., 2017). Furthermore, previous studies have shown evidence that low-frequency auditory information is important for lip reading (Breeuwer & Plomp, 1984; Faulkner, Ball, Rosen, Moore, & Fourcin, 1992). Studies of lip reading have found that tactile aids (with no accompanying audio) can improve the percentage of words correctly identified by around 9% for postlingually deafened adults, and by around 7% for normal-hearing listeners (Kishon-Rabin, Boothroyd, & Hanin, 1996). These studies typically included extensive training, of up to 300 h (e.g., Waldstein & Boothroyd, 1995). These findings indicate that another benefit of tactile stimulation in CI users may be enhanced lip-reading ability.

## Conclusions

This study has shown that tactile presentation of envelope and voicing information can improve speech-in-noise performance for normal-hearing subjects listening to CI simulations. This tactile enhancement effect was shown to increase substantially after just 30 min of exposure to speech-in-noise material and tactile stimulation over 3 days. The tactile signal was extracted from the speech-in-noise and presented via a single, small vibrating contact after computationally nonintensive signal processing. Real-time presentation of such tactile stimulation could be achieved by a compact, portable device and offer an inexpensive and noninvasive means for improving speech-in-noise performance in CI users.

## Appendix: Screening Questionnaire

Please fill in the following questionnaire to determine your eligibility for this experiment. If your answer ‘yes’ to any of the questions 4 to 14, please give additional details below. All data will be kept confidential.

1. What is your age in years? __________ Years
2. What is your gender? Male/Female
3. Are you right or left handed? Left/Right
4. Do you suffer from any conditions that might affect your sense of touch? Yes/No
5. Have you had any injury or surgery on your hands? Yes/No
6. Have you been exposed to severe or long periods of hand vibration? Yes/No
7. Have you had very recent exposure to hand vibration? Yes/No
8. Do you have a hearing impairment that you are aware of? Yes/No
9. Do you have, or have you recently had any pain, tenderness, infections, discharge, surgery or bleeding in either of your ears? Yes/No
10. Do you have a history of frequent exposure to loud noise? Yes/No
11. Do you take any ototoxic medications (for e.g., aminoglycoside antibiotics, such as gentamicin)? Yes/No
12. Do you experience tinnitus (ringing, buzzing, whistling or any other sounds in either of your ears)? Yes/No
13. Do you suffer from hyperacusis (reduced tolerance and increased sensitivity to everyday sounds)? Yes/No
14. Have you been exposed to loud sounds in the past 24 h? Yes/No If you have answered “yes” to any of the questions 4 to 14, please give further details below. Details for Question number (s): _____:
